# American Academy of Dental Science

**Published:** 1890-08

**Authors:** 

**Affiliations:** American Academy of Dental Science


### AMERICAN ACADEMY OF DENTAL SCIENCE.

### ADDRESS OF PROFESSOR F. W. PUTNAM BEFORE THE AMERICAN ACADEMY OF DENTAL SCIENCE AT THE PEABODY MUSEUM OF ARCHÆOLOGY AND ETHNOLOGY, CAMBRIDGE, JANUARY 8, 1890.

Mr. President and Gentlemen,—The principal object I have to-night is to call your attention to the importance of our collection of crania in a study of the diseases which affected the teeth and bones of the mouth of the people living in America at the time of, and prior to, the first occupation by the Europeans.

In our large collection, containing several hundred crania from various parts of the world, and, probably, over fifteen hundred from America, there is considerable valuable material for the study of various diseased conditions of the mouth, singular modifications of the palate, and anomalies in the position and shape of the teeth. I might have brought hundreds of crania to the lecture-room, all of which would have been interesting from your professional point of view, but I have limited myself to the few on the table, simply as examples of what you may expect to find if any of you wish to make a study of the whole collection.

Here are twenty skulls from the Santa Barbara Islands, off the coast of Southern California. In 1542 the people of these islands and from the main land came into contact with the Spaniards; and after the establishment of the missions on the coast they rapidly deteriorated, and finally succumbed to the changed conditions of life forced upon them by the whites, and to the vices which the contact brought about.

While many of the burials on these islands were long before white contact, others were after that period, as shown by the various objects of European work found in the graves. Hence we have here the opportunity of tracing the effect of the change of life which came to the people, and some of the marked cases of diseased conditions may be due to this change. Still it is evident that the primitive life of the people and their peculiar food had much to do with the singular wearing away of the teeth which is noticeable in this series. Starting with this skull, where every tooth is sound, you will notice the flat crowns of all, and the perfect occlusion. I think you have seldom seen a nearer approach to the movements of a jaw of a ruminant in a human subject than must have been the case in this man. Passing from this skull down the series as here arranged, you will notice the gradual wearing away of the teeth caused by the grinding of hard substances; this wear was probably increased by the sand mixed with the food, derived, partly, from pounding the seeds and acorns—which probably formed a constant diet—in stone mortars. As a result of this grinding process we can trace all the abscesses, and terrible suffering which the people must have endured, as shown in these jaws. Finally, nature gave relief to those who survived the suffering, by throwing off the roots of the teeth and closing the alveoli, as shown to be the case in so many of the crania, where, even before great age, the maxillaries were reduced to thin plates of bone.

So much has been said about syphilis in America, and that foul disease has been so often considered as of American origin, and responsible for many of the conditions met with in crania such as these, that I shall take this opportunity to say that, so far as can be determined by a study of the skeletons which we have, I consider there is not the slightest evidence of the occurrence of the disease in America until after contact with the whites. These skeletons have been studied by specialists, but in no case has any trace of the disease been found in skeletons antedating white contact. It is but just, therefore, to relieve our native American peoples from this foul imputation.

For comparison with the California crania I have chosen this skull of a Massachusetts Indian. This skull is from Winthrop, and from a burial-place which I have reasons for believing was not used after the first settlement of the whites at that place, at least two hundred and fifty years ago. This skull is of the same shape and character as the Californians, but you will notice that while this has a bad abscess in the under jaw, the teeth are not ground down in the same way as in the California crania. Here we probably have the evidence of a different diet and one containing not quite so much sand and gritty material.

In this series of skulls from the Sandwich Islands is probably the evidence of another kind of diet,—flesh and fruit. In the skulls the teeth are all sound and sharp and no sign of grinding or wear. Then, again, the jaws and palate are more like those of the white race; and, as you will notice, they have not the constant width of the Californians, but vary very much in shape.

Professor Putnam then called attention to a number of anomalies in the dentition of the several skulls on another table. They were from Peru and from the mounds of Ohio. In several of the Peruvian skulls the lateral growth of the third molar was pointed out, and in those from Ohio he referred to the probable later development of this tooth and its non-development in some instances. He also pointed out several instances of supernumerary teeth, and one skull where the left canine had turned laterally and posteriorly and penetrated the maxillary under the anterior edge of the malar bone.

As showing the resistance of enamel to natural decay, a mass of clay was shown which contained the enamel shells of a full set of teeth, while the skull and all other portions of the skeleton had been reduced to fine particles, or bone dust. This was from a very old burial near the Serpent Mound in Ohio. He had in several other instances found the enamel of the teeth the last part of the skeleton to decay. In some instances, however, he had found fragments of bones, even the spongy portions of the humerus, where there was no trace of other bones or of the enamel of the teeth. He could not say, therefore, that the enamel was always the last to decay, but thought this was likely to be the case with the skeletons of children.

DISCUSSION OF PROFESSOR PUTNAM'S ADDRESS.

*President Seabury.*—Gentlemen, we have all listened with great pleasure to this very interesting lecture of Professor Putnam. The matter is open for discussion or questions. I presume you have many questions which you would like to ask and which Professor Putnam will be glad to answer.

*Dr. Fillebrown.*—I would like to ask more particularly as to the age of these California skulls,—were they young people or adults? Has it been demonstrated that they when young had cusps that were equally prominent with other races?

*Professor Putman.*—On that point you will have to put the facts together and draw your own conclusions. You will find that the teeth are very large. Now, it is beyond the power of the average investigator to get at the exact age of the individuals, but judging by the sutures,—which we go by in determining the age more than by any development of the teeth, because we cannot assume the protrusion of the teeth in other races at the same years as in the white race,—all the skulls on the table are fully adult and many of advanced age.

*Dr. Fillebrown.*—In looking over the skulls of these Californians, I should say that the cusps of the teeth of this people were less prominent than in the Sandwich Islanders, and that the occlusion of the teeth has more nearly the characteristic of the ruminant, and that the overhanging of the upper jaw is less pronounced than in the races of the present day. To-day we consider it an exception where the teeth occlude all round; but here the indications are that it was a race characteristic.

*Professor Putnam.*—I think it will be found to be common to many peoples other than the white race.

*Dr. Fillebrown.*—There is one more question which I should like to ask Professor Putnam, and that is, whether or not there are some indications by which you can determine the sex? The appearance of that Roman skull seems to indicate its being the skull of a female. In the first place, the bones seem to me to be thinner, and secondly, we see smaller teeth and more delicate ones in every way, and especially I notice the characteristic of the lower jaw that seemed to indicate to me that it was the jaw of a female.

*Professor Putnam.*—Taking all the male and female characteristics into consideration, I should regard the skull in question as that of a woman. I suppose it is hardly fair to compare a Roman lady with a California Indian, but we will do so in this case for the sake of contrasting her skull with this skull, which has all the male characteristics so strongly marked that you can see the differences at once.

*Dr. Fillebrown.*—I trust Professor Putnam will not consider that we are boring him, but I am so much interested in the comparison of the skulls here, and as it is a line of observation that I have not been familiar with before,—my own observations having been confined to the upper jaw,—that it would please me very much, and I believe others also, if he would show us the comparison of the sexes more thoroughly and explain what are the distinctive characters of the males and females.

In reply, Professor Putnam pointed out many differences which are considered as sexual, and stated that the differences between the sexes were not so marked among savage peoples as among civilized.

*Dr. Fillebrown.*—I have just one more thought to present, and that is a conclusion I received from the hint that Professor Putnam gave us to-night. I have thought of this before, but it has more clearly than ever appeared to me to-night. It has been claimed that a typical set contains forty-four teeth. The transition from the human incisor to the cuspid, and from the cuspid to the first bicuspid, also from the second bicuspid to the first molar, is very marked. I understand the theory is that one incisor and a first and fourth bicuspid have been lost in the development of the human type. Professor Putnam's observations seem to indicate that when the more perfect man shall arrive, a few thousand years hence, the third molar will be obliterated.

*Dr. Andrews.*—I would say that a former patient of mine had the fourth molar, thirty-six teeth in all. They did not cause any inconvenience, nor have the appearance of an anomaly, but they were as beautiful a set of natural teeth as I ever saw.

*Dr. Fillebrown.*—I have a model of a mouth that I took where the patient had three well-developed bicuspids.

*Dr. Andrews.*—On both sides of the jaw, upper and under?

*Dr. Fillebrown.*—I do not remember about the under jaw, but on both sides of the upper jaw there were three fully developed bicuspids.

*Dr. Andrews.*—Mr. President, I move that the privilege of the floor be allowed to all those present.

*The President.*—Without the formality of a vote, I know that the privilege will be accorded to any one wishing to speak. We shall be happy to hear from any gentleman present.

*Dr. Codman.*—There is one thing it seems to me has been brought out this evening, and that is, that similar causes bring about similar results. Here are the skulls of a race of people who lived in a certain way, and their teeth are worn down accordingly. If they had lived in another way, if they had another diet and other habits, the development of the wear of the teeth would have been different. Diet has a very strong influence in the wear of the teeth.

*Dr. Taft.*—One thing that I noticed, especially in the teeth of the lower jaw that were passed around, was the peculiar wearing away from the lingual to the buccal side almost down to the gums. I would like to ask Professor Putnam whether that comes so much from the diet as it does from the occlusion, which so closely resembles that of the ruminants?

*Professor Putnam.*—I could not say; it does exist throughout all the skulls of these Californians.

*Dr. Taft.*—You spoke particularly of their diet consisting of acorns and roots.

*Professor Putnam.*—Yes; it must certainly have had some effect.

*Dr. Fillebrown.*—There is another thought that occurs to me in regard to the appearance of these teeth. If any one takes food into their mouth upon the right side, they will be sure to move the jaw towards the left, and *vice versa.* Consequently the bite comes upon the inner cusps of the upper teeth, and the outer cusps of the under teeth. That will account for the wear of all the teeth of these skulls.

*The President.*—If there is nothing more to say, we will pass the subject.

*Dr. Fillebrown.*—Mr. President, I think at this time it would be very appropriate, and I move you, sir, that the hearty thanks of this society be tendered to Professor Putnam for his very interesting lecture before the society.

*Professor Putnam.*—Mr. President, in accepting this vote of thanks, I wish to say that this museum was founded for the study of anthropology; that it has been the work of years of earnest endeavor to make collections which will give the means of studying man in all his various conditions and habits. We wish to have this museum made use of, and I hope that you will avail yourselves of the facilities it offers.

William H. Potter, D.M.D.,
*Editor American Academy of Dental Science.*

